# Supplementary material for: Enapotamab Vedotin, an AXL-Specific Antibody-Drug Conjugate, Demonstrates Antitumor Efficacy in Patient-Derived Xenograft Models of Soft Tissue Sarcoma
Source: Int J Mol Sci. 2022 Jul 6;23(14):7493. doi: 10.3390/ijms23147493 (PMC9322120; doi:10.3390/ijms23147493)
Supplement: Supplementary file 1 [file ijms-23-07493-s001.zip › ijms-1772476-supplementary.pdf]

Table S1. Detailed description on the number of mice/tumors (n) included in tumor volume (and survival) evaluation, average absolute tumor volume (and range) and length of each experiment.

Xenograft	Isotype control ADC		EnaV		Last day of observation
	(n)	Average absolute tumor volume and range (mm^3)	(n)	Average absolute tumor volume and range (mm^3)	
UZLX-STS3^{DDLPS}	9 (6)	249 (43 -800)	9 (6)	258 (67 -858)	64
UZLX-STS124^{DDLPS}	8 (5)	75 (46 – 100)	10 (7)	96 (33 -205)	103
UZLX-STS204^{DDLPS}	10 (7)	147 (98 – 226)	10 (7)	165 (115 – 236)	82
UZLX-STS81^{LMS}	7 (4)	201 (111 – 345)	9 (6)	248 (52 – 857)	76
UZLX-STS128^{LMS}	6 (3)	155 (101 – 213)	8 (5)	129 (39 – 281)	78
UZLX-STS126^{MFS}	9 (6)	303 (181 – 485)	9 (6)	280 (160 – 419)	99
UZLX-STS132^{MFS}	10 (7)	456 (178 -668)	10 (7)	415 (201 – 587)	101
UZLX-STS84^{UPS}	8 (5)	237 (58 – 431)	9 (6)	270 (78 -504)	85

DDLPS: dedifferentiated liposarcoma; LMS: leiomyoscarcoma; MFS: myxofibrosarcoma; UPS: undifferentiated pleomorphic sarcoma

Table S2. Summary of results from tumor volume evaluation, representing the average relative tumor volume ± standard deviation (%) of each xenograft experiment at day 22.

Xenograft	Isotype control ADC	EnaV	Unpaired t-test	Paired t-test	Log-rank test
UZLX-STS3^{DDLPS}	660 ± 329	255 ± 103	p=0.006	p=0.004	p=0.857; HR (95% CI)=1.093 (0.352 - 3.393)
UZLX-STS124^{DDLPS}	341 ± 157	193 ± 118	p=0.036	p=0.034	p=0.114; HR (95% CI)=3.416 (0.651 – 17.930)
UZLX-STS204^{DDLPS}	162 ± 126	90 ± 82	p=0.140	/	p=0.620; HR (95% CI)=1.500 (0.260 – 8.659)
UZLX-STS81^{LMS}	657 ± 242	555 ± 238	p=0.444	/	p=0.325; HR (95% CI)=1.743 (0.436 – 6.966)
UZLX-STS128^{LMS}	317 ± 137	16 ± 9	p=0.000	p<0.000	p=0.116; HR (95% CI)=9.439 (0.574 – 155.200)
UZLX-STS126^{MFS}	102 ± 32	106 ± 45	p=0.931	/	p=0.001; HR (95% CI)= 21.68 (3.630 – 129.500)
UZLX-STS132^{MFS}	124 ± 53	100 ± 72	p=0.413	/	p=0.055; HR (95% CI)=6.169 (1.026 – 37.110)
UZLX-STS84^{UPS}	1273 ± 631	52 ± 36	p<0.000	p=0.004	p=0.003; HR (95% CI)=26.27 (2.974 – 232.100)

DDLPS: dedifferentiated liposarcoma; HR: hazard ratio; LMS: leiomyoscarcoma; MFS: myxofibrosarcoma; UPS: undifferentiated pleomorphic sarcoma; /: not done; 95% CI: 95% confidence interval.

Table S3. Explanation on dropouts other than tumor volume > 2000 mm^3, observed during the experiment.

Xenograft	Treatment group (day)	Remarks
UZLX-STS84^{UPS}	Isotype control ADC (day 15)	Severe body weight loss (n=1)
	EnaV (day 47)	Found dead (n=1)
	EnaV (day 50)	Found dead (n=1)
	EnaV (day 64)	Found dead (n=1); severe body weight loss (n=1)
	EnaV (day 85)	Found dead (n=1)
	EnaV (day 92)	Found dead (n=1)
UZLX-STS124^{DDLPS}	EnaV (day 40)	Found dead (n=1)
UZLX-STS126^{MFS}	Isotype control ADC (day 53)	Tumor ulceration (n=2)
	Isotype control ADC (day 64)	Tumor ulceration (n=2)
	Isotype control ADC (day 85)	Tumor ulceration (n=2)
UZLX-STS128^{LMS}	Isotype control ADC (day 72)	Severe body weight loss (n=1)
	EnaV (day 8)	Severe body weight loss (n=1)
	EnaV (day 37)	Found dead (n=1)
UZLX-STS132^{MFS}	Isotype control ADC (day 38)	Severe body weight loss (n=1)

DDLPS: dedifferentiated liposarcoma; LMS: leiomyoscarcoma; MFS: myxofibrosarcoma; UPS: undifferentiated pleomorphic sarcoma.

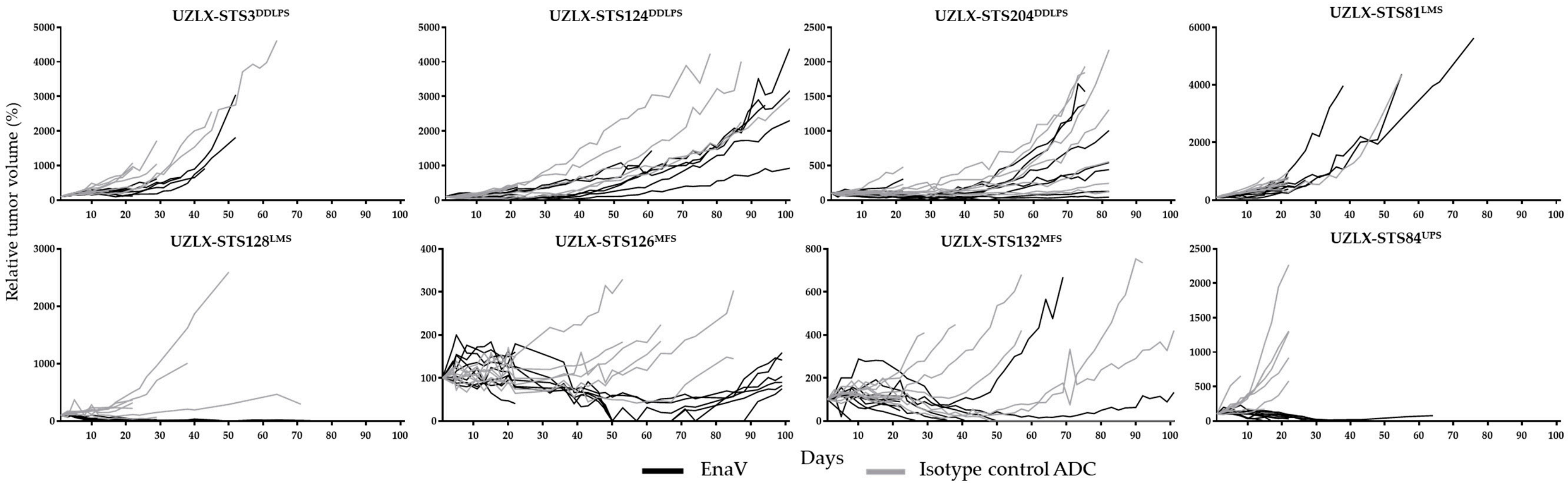

Figure S1. Individual relative tumor growth curves of each xenograft experiment.

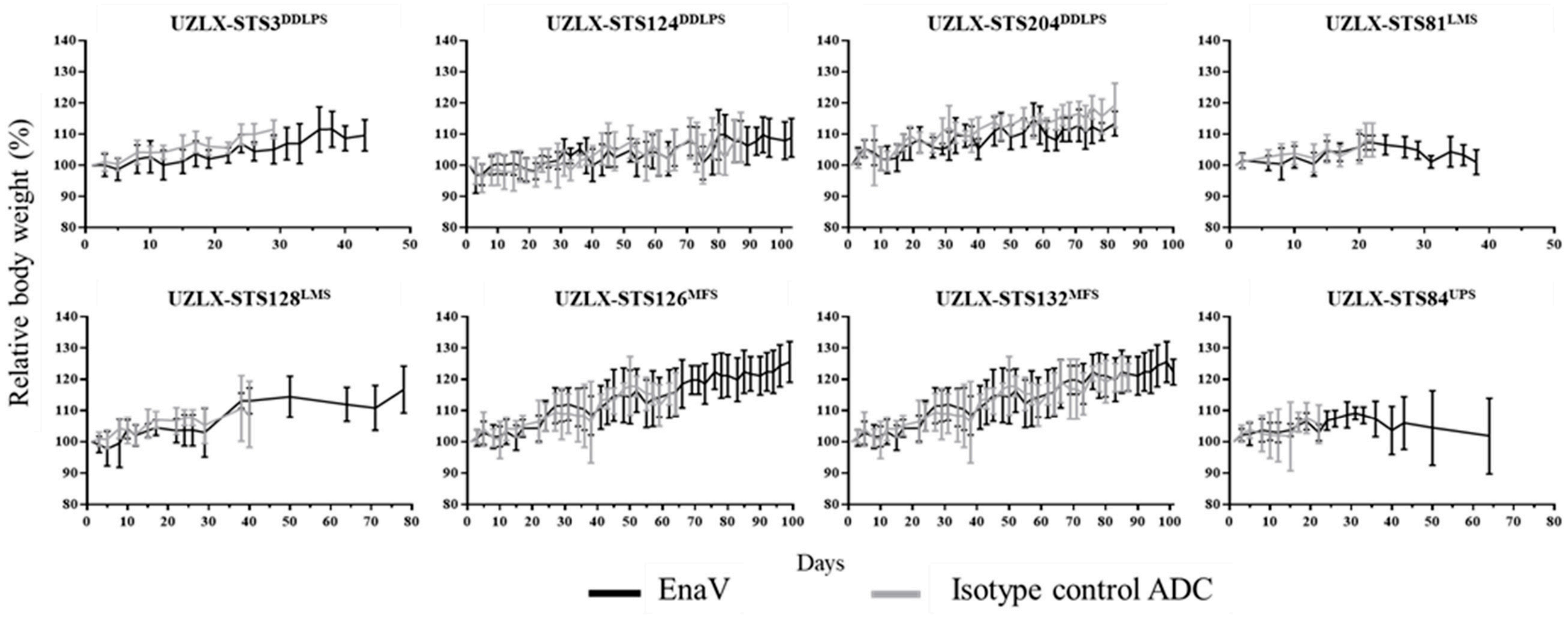

Figure S2. Relative body weight evaluation of the EnaV-treated patient-derived sarcoma xenografts, demonstrated as average ± standard deviation (%) of at least three animals.

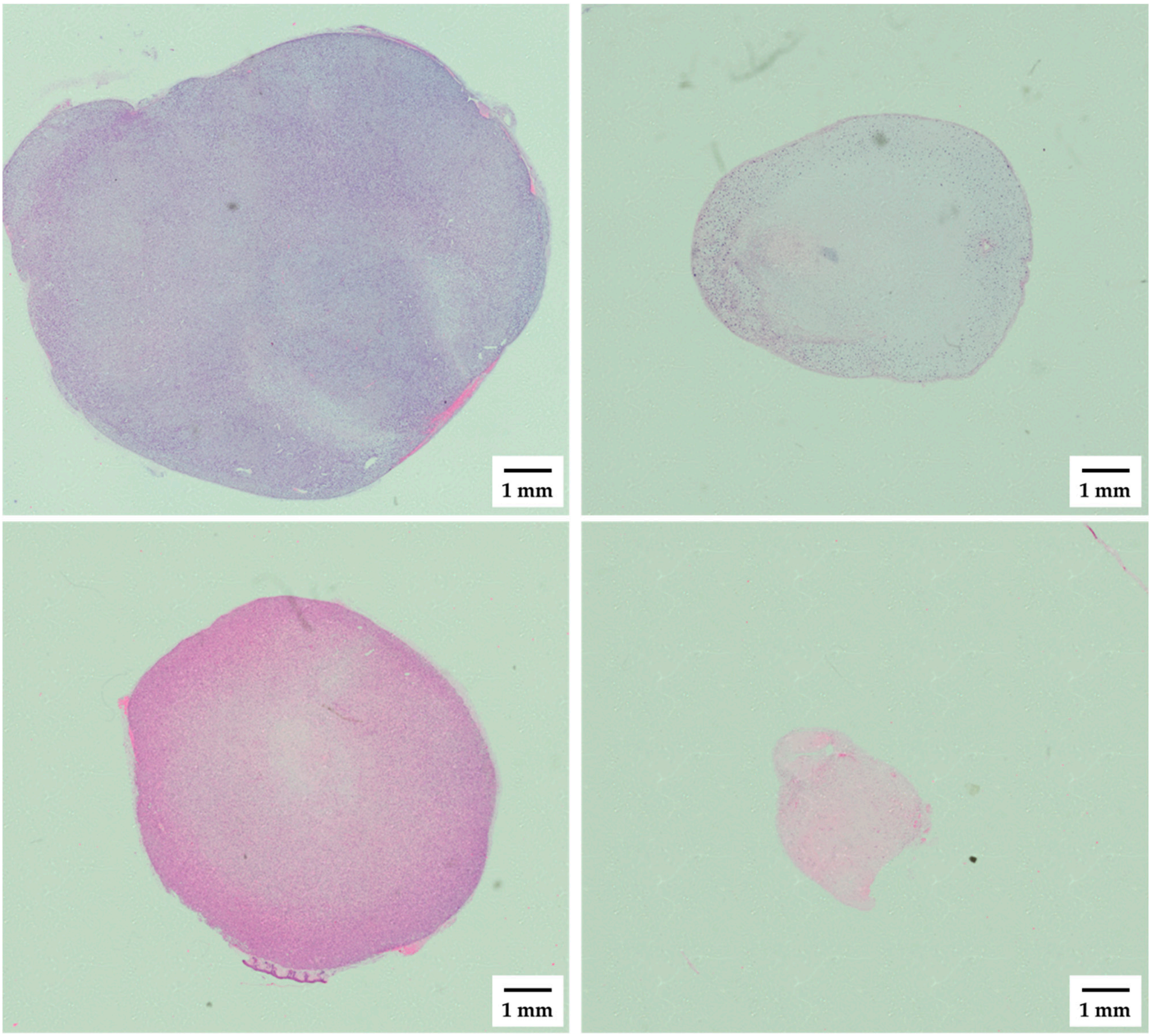

Figure S3. Representative scans of tumors from UZLX-STS84^{UPS} (upper) and -STS128^{LMS} (lower) collected at day 22 of the experiment.
